# Book Reviews

**Published:** 1895-06

**Authors:** 


### *BOOK REVIEWS.*

---

Clinical Gynecology, Medical and Surgical. For Students and Practitioners. By Eminent American Teachers. Edited by John M. Keating, M.D., LL.D., and Henry C. Coe, M.D., M.R.C.S. Philadelphia. J. B. Lippincott Company. 1895.

Works on gynecology have multiplied rapidly of late. The constant progress made in this special line of work is to some extent a justification and to a very great extent the excuse for such rapid production. The work before us occupies a somewhat different field and fills a somewhat different want from its predecessors. It aims, and aims successfully, to represent the gynecology of to-day, the up-to-date status of this branch of medicine. It is not the work of one mind, but of many.

A glance at the list of writers who have furnished contributions is sufficient to inspire confidence from the start. They are all progressive, active thinkers and workers in gynecology, men who have already won their spurs, and who write from an experience sufficiently large to give weight and value to their opinions. Prominence is given to the practical rather than the theoretical side of the subject, as is eminently proper in a work of this kind.

Much of the matter is of necessity a repetition of what has already been said in other books. The established facts of gynecology are now numerous enough to make up a large mass of material which will permanently remain essentially unchanged. One of the best chapters in the book, because it is new and relates to fundamental principles, is that upon "Gynecological Technique," by Hunter Robb. Nowhere except in the monograph, by the same author, can so complete an exposition of the subject be found. The chapter by Polk upon "Inflammation of the Female Genital Organs" is marked by the thoroughness and excellence which characterize all the productions of this able writer. In the chapter on "Displacements of the Uterus," the author (Mundé) starts out by saying that displacements (with the exception of inversion)

possess very little significance *per se*, and then devotes sixty-six pages to their consideration. With the exception of the preliminary statement, this chapter might as well perhaps have been left out. The chapter upon "Neoplasms of the Ovaries, Tubes, and Broad Ligaments" is a complete monograph in itself. The fact that it is from the pen of Henry C. Coe is sufficient guarantee of the thorough care with which the subject is elaborated. The chapter on "Diseases of the Bladder" gives a full description of Kelly's recent valuable improvements in cystoscopy. All the other parts of the book are good, but lack of space forbids their specific mention. Suffice it to say, that the physician will make no mistake who adds this volume to his library and makes it one of his books of every-day reference.

---

TWENTIETH CENTURY PRACTICE. An International Encyclopedia of Modern Medical Science. By Leading Authorities of Europe and America. Edited by Thomas L. Stedman, M.D., New York City. In twenty volumes. Volume II. Nutritive Disorders. New York: William Wood & Company. 1895.

Sir Dyce Duckworth, of London, contributes the first article in this volume, a description of "Addison's Disease and Other Diseases of the Adrenals." The next one hundred and fifty pages are devoted to "Diabetes Mellitus," by Professor von Noorden, of Frankfort. This article is very complete and practical; sixty pages being devoted to treatment, special, hygienic, and dietetic. The articles on "Rheumatism" and "Gout" are the best and most interesting in the book. The former is by Dr. T. J. Maclagan, of London, who introduced (1876) the anti-rheumatic treatment by the salicyl compounds. The author is a believer in the malarial causation of rheumatism (p. 228). Dr. Henry M. Lyman, of Chicago, contributes the paper on Gout. This article is nothing less than a comprehensive treatise on this disease, occupying one hundred and eighty pages. Dr. A. E. Garrod, of London, describes "Arthritis Deformans" (sixty pages). The article on "Diseases of the Muscles," by Dr. Dujardin-Beaumetz, of Paris, is not as complete as it might be. This article was almost the last literary work completed by the distinguished author before his death. Professor

Oertel, of Munich, contributes the article on "Obesity" (one hundred pages). The author's method of treating obesity, now so generally practiced, is fully described and its *rationale* explained.

All of the articles in this volume have been written by master hands, and are full of interest and instruction for every reader.

---

MATERIA MEDICA AND THERAPEUTICS. For Physicians and Students. By John B. Biddle, M.D., late Professor of Materia Medica and Therapeutics in the Jefferson Medical College, Philadelphia. Thirteenth edition. By Clement Biddle, M.D., Medical Corps U. S. Navy. P. Blakiston, Son & Co., 1012 Walnut street, Philadelphia.

A work which has enjoyed the popularity of this one, and which has passed through thirteen editions since 1865, requires little more to be said of it than that the last edition maintains the high order of its predecessors, besides taking full cognizance of recent progress in materia medica and therapeutics. This edition has been made to conform to the 1890 U. S. Pharmacopeia, containing all the preparations and remedies described therein. Certain new remedies have been introduced, as alumnol, creolin, lysol, salipyrin, piperazine, and an appendix includes a number of remedial agents now on probation. The book is complete and well up to date, and will be found a satisfactory work of reference to the physician and an excellent text-book for the student.

---

DOSE-BOOK AND MANUAL OF PRESCRIPTION WRITING. By E. Q. Thornton, M.D., Ph.G., Demonstrator of Therapeutics, Jefferson Medical College, Philadelphia. Price, $1.25. W. B. Saunders, 925 Walnut street, Philadelphia.

A book of this sort is almost essential to the student and young practitioner. This one is about as good as any which we have ever seen, and, in some respects, superior to most others. The part devoted to prescription writing is especially clear and complete. The book contains a full list of official drugs and their preparations, including the most important new remedies, and their doses in both English and metric systems. Where a book of this sort is wanted for study or reference, we think this will be entirely satisfactory.

